# COX-2 inhibitor celecoxib prevents chronic morphine-induced promotion of angiogenesis, tumour growth, metastasis and mortality, without compromising analgesia

**DOI:** 10.1038/sj.bjc.6604057

**Published:** 2007-10-30

**Authors:** M Farooqui, Y Li, T Rogers, T Poonawala, R J Griffin, C W Song, K Gupta

**Affiliations:** 1 Division of Hematology, Oncology and Transplantation, the Vascular Biology Center, Department of Medicine, University of Minnesota, Minneapolis, MN 55455, USA; 2 Department of Therapeutic Radiology, University of Minnesota, Minneapolis, MN 55455, USA; 3 Breast Cancer Research Program, Department of Radiation Oncology, University of Arkansas for medical Sciences, Little Rock, AR 72205, USA

**Keywords:** opioids, morphine, angiogenesis, cyclooxygenase-2, breast cancer, metastasis

## Abstract

Morphine and its congener opioids are the main therapy for severe pain in cancer. However, chronic morphine treatment stimulates angiogenesis and tumour growth in mice. We examined if celecoxib (a cyclooxygenase-2 (COX-2) inhibitor) prevents morphine-induced tumour growth without compromising analgesia. The effect of chronic treatment with celecoxib (by gavage) and/or morphine (subcutaneously), or PBS on tumour prostaglandin E_2_ (PGE_2_), COX-2, angiogenesis, tumour growth, metastasis, pain behaviour and survival was determined in a highly invasive SCK breast cancer model in A/J mice. Two weeks of chronic morphine treatment at clinically relevant doses stimulates COX-2 and PGE_2_ (4.5-fold compared to vehicle alone) and angiogenesis in breast tumours in mice. This is accompanied by increased tumour weight (∼35%) and increased metastasis and reduced survival. Co-administration of celecoxib prevents these morphine-induced effects. In addition, morphine and celecoxib together provided better analgesia than either agent alone. Celecoxib prevents morphine-induced stimulation of COX-2, PGE_2_, angiogenesis, tumour growth, metastasis and mortality without compromising analgesia in a murine breast cancer model. In fact, the combination provided significantly better analgesia than with morphine or celecoxib alone. Clinical trials of this combination for analgesia in chronic and severe pain in cancer are warranted.

Opioids are the mainstay of treatment for severe pain in advanced cancer, including metastatic breast cancer (Levy, 1996; Mantyh, 2006). Although opioids act directly on the central nervous system to relieve pain, their activities on peripheral tissues are responsible for many of the secondary complications associated with the management of cancer pain. A recently recognised peripheral effect of opioids and their receptors is the promotion of angiogenesis-dependent tumour growth. Opioids at physiologically relevant concentrations promote angiogenesis *in vitro*, and in breast cancer and wound healing in rodents (Gupta *et al*, 2002; Poonawala *et al*, 2005; Singleton *et al*, 2006). Although naloxone and naltrexone can inhibit tumour growth in rodents (Zagon and McLaughlin, 1983a; Koo *et al*, 1996; Gupta *et al*, 2002), opioid receptor antagonists cannot be used to counteract the unwanted effects of opioids without also compromising analgesia in the clinical setting. Therefore, it is important to identify agents that spare the analgesic effect of opioids while inhibiting their angiogenic effect.

One key mechanism of morphine-induced angiogenesis is nitric oxide (NO)-dependent MAPK phosphorylation and endothelial proliferation (Gupta *et al*, 2002; Poonawala *et al*, 2005). Morphine stimulates NO in endothelium and chronic morphine treatment increases NO synthase (NOS), NO and cyclooxygenase-2 (COX-2) in mouse kidney (Stefano *et al*, 1995; Arerangaiah *et al*, 2007). Nitric oxide stimulates the enzymatic activity of COX (Salvemini *et al*, 1993), and activated COX in turn increases prostaglandin E_2_ (PGE_2_) production (Salvemini *et al*, 1993, 1994; Nedelec *et al*, 2001; Birnbaum *et al*, 2005). Prostaglandin E_2_ promotes angiogenesis and tumour progression, and blockade of COX-2 activity inhibits angiogenesis and tumour progression (Griffin *et al*, 2002; Leahy *et al*, 2002; Chang *et al*, 2004). On the other hand, prostaglandins are also implicated in processing pain (Malmberg and Yaksh, 1992; Julius and Basbaum, 2001; Samad *et al*, 2001). Indeed, COX-2 inhibitors inhibit the development of morphine tolerance (Wong *et al*, 2000).

It is likely that the products of COX-2 such as PGE_2_ produced in tumour cells act on the tumour endothelium and promote angiogenesis. Therefore, morphine-induced tumour growth may in part be due to the upregulation of COX-2. We hypothesised that the prolonged use of opioids in cancer stimulates COX-2 and PGE_2_, leading to angiogenesis-dependent tumour progression and metastasis and mortality. This raises the possibility that highly selective COX-2 inhibitors such as celecoxib may block morphine-induced angiogenesis and tumour growth without compromising analgesia. We examined the effects of celecoxib on opioid-induced COX-2 expression, pain, angiogenesis, tumour progression, metastasis and survival in a murine breast cancer model. We demonstrate that celecoxib prevents opioid-induced tumour progression and metastasis and promotes survival, apparently without impairing the analgesic effect of opioids.

## MATERIALS AND METHODS

### Animals

Eight- to twelve-week-old A/J mice (The Jackson Laboratory, Ann Harbor, ME, USA) were maintained under controlled environmental conditions (12-h light to dark cycle, at 23°C) in our AAALAC-accredited facility and provided with standard laboratory food and water *ad libitum*. All experiments were performed according to the protocols approved by the Institutional Animal Care and Use Committee (IACUC) at the University of Minnesota. Mice were euthanised using compressed CO_2_ from the gas cylinders at the conclusion of the duration of the treatment and/or if they appeared moribund (unable to reach food and water and impaired mobility).

### Drugs and chemicals

Pharmacological-grade morphine (Baxter Esilerderle Mfd. Healthcare Corporation, Cherry Hill, NJ, USA), and Celecoxib (celecoxib capsules, Pfizer Inc., New York, NY, USA) were used. All other chemicals were obtained from Sigma Chemicals (St Louis, MO, USA), and cell culture reagents were obtained from Invitrogen unless otherwise mentioned.

### Tumour model

SCK mammary carcinoma was generated in rodents using breast cancer cells derived from a tumour that spontaneously arose in a female A/J mouse (Jackson Laboratories) in our laboratory (Griffin *et al*, 2002). These tumours grow to ∼1000 mm^3^ in 12 days, and they are highly vascular, invasive and metastasise to the lungs, resulting in the death of the animals starting at about 15 days after tumour-cell injection. SCK tumour cells (2 × 10^5^) in 0.05 ml of serum-free medium were injected subcutaneously into the right hind thigh of A/J mice. Palpable and measurable tumours appeared 4–5 days after injection.

### Treatment groups

Tumour-bearing mice were randomly assigned to experimental groups 1 day after tumour cell injection. Treatments for each experiment were administered twice daily.

#### Protocol I

To examine the effect of celecoxib on tumour PGE_2_, angiogenesis, tumour growth and survival, mice were equally divided into four groups of (a) normal saline s.c. at 50 *μ*l and 0.5% methylcellulose at 100 *μ*l by gavage (control); (b) morphine sulphate s.c. at 0.714 mg per kg per mouse per day for 7 days and 1 mg per kg per mouse per day thereafter (equivalent to 50 and 75 mg of morphine per day for a 70 kg human, respectively) and 0.5% methylcellulose at 100 *μ*l by gavage to ensure similar administration of vehicle in each animal; (c) celecoxib at 30 mg per kg per mouse by gavage in 0.5% methylcellulose and (d) morphine sulphate plus celecoxib as described in b and c.

#### Protocol II

To determine the effect of high-dose celecoxib, mice were treated with (a) normal saline s.c. at 50 *μ*l and 0.5% methylcellulose at 100 *μ*l by gavage; (b) morphine sulphate s.c. at 0.714 mg per kg per mouse per day for 7 days and 1 mg per kg per mouse per day thereafter; (c) celecoxib at 100 mg per kg per mouse by gavage in 0.5% methylcellulose and (d) morphine sulphate plus celecoxib as described in IIb and IIc.

### Tumour neovascularisation

Tumour cryosections were immunostained with the endothelial cell marker anti-CD31-PE (1 : 50 dilution; Pharmingen, San Diego, CA, USA) and the nuclear stain DAPI (Molecular Probes, Eugene, OR, USA), as described previously (Poonawala *et al*, 2005). Three different sections of tumour were selected for image analysis, and digital images of at least three different areas of each section were binarised and linearized to quantify total PE-positive pixels in each image as a measure of such angiogenic parameters as blood vessel length, ends and nodes, using the Image Processing Tool kit, Plug-in Functions for Adobe PhotoShop (Reindeer Games, Asheville, NC, USA), as described before (Gupta *et al*, 2002).

### Behavioral analysis

#### Thermal escape latency

Tumour-bearing mice were acclimated on the glass surface of the chamber of the Paw Thermal Stimulator System (UCSD, San Diego, CA, USA) for 60 min before the test (Hargreaves *et al*, 1988). A radiant heat stimulus was applied to the plantar surface of a single hind paw from underneath the glass floor with a projector lamp bulb (CXL/CXR, 8 V, 50 W). Paw withdrawal latency (PWL) to the nearest 0.1 s was automatically recorded when a mouse withdrew its paw from the stimulus. Stimulus intensity was adjusted to derive an average baseline PWL of approximately 10.0 s in PBS-treated control mice. A 20-s stimulus cutoff was used to prevent tissue damage.

### Western blot analysis

Whole tumour lysates containing 50 *μ*g of protein were resolved on 3–15% gradient SDS-PAGE gels, followed by transfer to the PVDF membrane (Immobilion, Millipore, Bedford, MA, USA). Membranes were blocked for 1 h at room temperature in 5% non-fat dry milk in Tris-buffered saline with 0.1% Tween 20, and incubated with 1 : 500 dilution of anti-COX-2 antibody (Cayman Chemical, Ann Arbor, MI, USA) or anti-*β*-actin (Santa Cruz Biotechnology Inc., Santa Cruz, CA) at 4°C overnight. After washing, membranes were incubated with 2° species-specific antibodies conjugated to alkaline phosphatase for 45 min at room temperature. Proteins were visualised with an ECF Western blotting system (Amersham Life Sciences, Buckinghamshire, UK). Chemiluminescent signals were acquired using a Storm 860 PhosphorImager (Molecular Dynamics, Sunnyvale, CA, USA).

### RT-PCR

Total RNA was isolated from tumours using Trizol reagent (Farooqui *et al*, 2006). Five micrograms of total RNA was reverse transcribed using a First-Strand Synthesis System (Invitrogen, Carlsbad, CA, USA). PCRs were performed by using Taq DNA polymerase (Continental Lab Products, San Diego, CA, USA). Sequences of primers homologous to the coding region of mouse COX-2 (GenBank acc. no. NM_011198) were 5′-ACTCACTCAGTTTGTTGAGTCATT-3′ (sense nucleotides 1312–1335) and 5′-TTTGATTAGTACTGTAGGGTTAAT-3′ (antisense nucleotides 1871–1894); sequences for mouse GAPDH (GenBank acc. no. NM_001001303) were: 5′-CGTCTTCACCACCATGGAGA-3′ (sense nucleotides 353–371) and 5′-CGGCCATCACGCCACAGTTT-3′ (antisense nucleotides 635–651) (Lim *et al*, 2006). Amplification was performed for 30 cycles at 94°C for 50 s, 56°C for 50 s and 72°C for 50 s with a final extension cycle for 10 min at 72°C in a PTC-100 thermocycler (MJ Research, Waltham, MA, USA). DNA samples were separated by electrophoresis in 2% agarose gel. The purified PCR products obtained were sequenced (Microchemical Facility, University of Minnesota) to verify that they matched the expected DNA sequences.

### Prostaglandin E_2_ assay

Prostaglandin E_2_ levels were estimated in whole tumour lysates using an EIA Kit (Cayman Chemical Company, Ann Arbor, MI, USA) as per the manufacturer's instructions. Lysates were purified using acetone precipitation of proteins, and supernatants from each sample were quantified using two different dilutions of each. In this method, the intensity of the colour produced is measured at 412 nm and is inversely proportional to the amount of PGE_2_ present in the well. Recovery of PGE_2_ was calculated based on the recovery of a cold spike of PGE_2_ added to the lysis buffer and processed in parallel with tumour lysates. Prostaglandin E_2_ content was calculated using a calibration curve prepared with 10–1000 pg per ml of PGE_2_.

### Histology

Lungs were fixed in 10% formalin overnight, and paraffin-embedded lung sections were stained with haematoxylin and eosin. Metastatic foci were visualised in a double-blinded manner using a light microscope.

### Statistical analysis

Duration of survival in the four treatment groups was analysed with Kaplan–Meier curves. Equality of survival curves was assessed using the Wilcoxon's log-rank test, which weights differences in the curves by the number of surviving mice (Klein and Moeschberger, 1997).

Tumour weight was analysed with an ANOVA model, and pairwise comparisons among the four treatment groups were made using Tukey's studentised range test (HSD) (Christensen, 2002).

Four measures of vascular complexity (pixels, nodes, ends and line length) were performed at three sites in three mice from each treatment group. Mice were randomly selected from among those living at the end of 14 days. Differences between treatment groups of these measurements were assessed using an ANOVA model with a random intercept for each mouse to account for within-mouse correlation of measurements. Tests for treatment group effects used the between-within method for determining the degrees of freedom and maximum likelihood to fit the model (Fitzmaurice *et al*, 2004).

We assessed longitudinal changes in pain using a repeated-measures ANOVA model. The model includes three fixed-effect terms of treatment group, day, and treatment group by day interaction. It also includes a random-effect term for the mouse-specific baseline. Tests for treatment group effects at each day used the between-within method for determining the degrees of freedom and maximum likelihood to fit the model.

## RESULTS

### Morphine upregulates COX-2 expression and PGE_2_ in tumours

**Figure 1:**
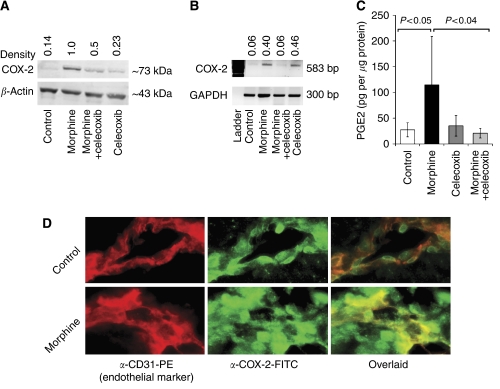
Cyclooxygenase-2 expression and PGE_2_ concentration in breast tumours of mice after 13 days of treatment (or 14 days after tumour cell injection) with morphine and co-administration with celecoxib. (**A**) Western blot showing upregulation of COX-2 protein in the region of 72–74 kDa, whereas *β*-actin is shown as a loading control. The arbitrary densitometric units above the bands show the ratio of COX-2 to *β*-actin. (**B**) RT-PCR, showing an upregulation in COX-2 gene expression in the expected region of 583 bp. Bands on the bottom show the equal loading of RNA represented by GAPDH. Arbitrary numeric units show the ratio of COX-2 to GAPDH expression. (**C**) Prostaglandin E_2_ concentration is significantly increased in tumours of mice treated with morphine. Data are shown as mean±s.d. *n*=3–5 experiments per observation/data shown. (**D**) Immunofluorescent staining of tumour cryosections using anti-CD31 PE (red), an endothelial cell marker and anti-COX-2 followed by a 2° Ab conjugated with FITC (green). The overlaid image in the right hand panel shows yellow staining due to increased COX-2 in the endothelium in morphine-treated mice. Larger number of non-endothelial cells show COX-2 expression (green) in middle and overlaid panel after morphine treatment. Magnification, × 600.

To determine whether morphine treatment exaggerates the expression of COX-2, we evaluated COX-2 protein and mRNA expression in SCK mammary tumours in A/J mice after 13 days of treatment using Protocol I. Cyclooxygenase-2 protein and mRNA levels were increased by five- to seven-fold after morphine treatment as compared to PBS-treated mice (Figure 1A and B). Upregulation of COX-2 expression was higher in endothelial and tumour cells in morphine-treated mice compared to PBS-treated controls, based on immunofluorescent staining of tumour cryosections (Figure 1C). Notably, morphine induced a much stronger expression of COX-2 in tumour cells than in endothelial cells. The upregulation of COX-2 protein and gene expression was accompanied by an ∼5-fold increase in PGE_2_ (*P*<0.05 *vs* control) in tumours of mice treated with morphine (Figure 1D). Co-administration of celecoxib blocked this morphine-induced increase in COX-2 expression and PGE_2_.

### Celecoxib inhibits morphine-induced tumour angiogenesis

**Figure 2:**
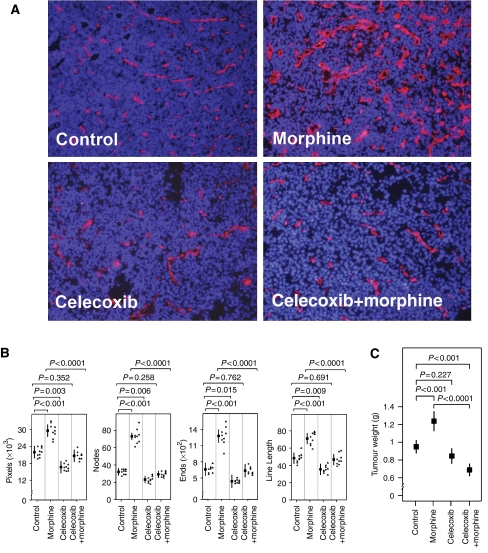
Effect of morphine and co-administration with celecoxib on tumour angiogenesis and tumour growth, after 13 days of treatment. (**A**). Tumour cryosections were stained with endothelial cell-specific anti-CD31 PE (red) and nuclear stain, DAPI (blue). The red and blue images of the same area are overlaid to show the tumour vasculature in red and cellular presence represented by blue nuclei. Note the dense, heavily branched and wide blood vessels distributed uniformly in tumours from morphine group. Celecoxib-treated mouse tumours exhibit fewer and collapsing blood vessels and areas with week CD31 staining suggesting degenerating vasculature. This week CD31 staining is accompanied by bald areas without any blue nuclei. However, celecoxib given with morphine did not result in degenerating looking vessels, even though the blood vessels were fewer, less dilated, smaller and accompanied by bald areas devoid of blue nuclei compared to morphine-alone-treated mice. Magnification, × 150. Each figure represents nine sections from three different mouse tumours. (**B**) Quantitative expression of vasculature in tumour sections is shown as PE-positive pixels (representing red staining for blood vessels), number of nodes (equivalent to branch points), vessel ends (for the number of vessels) and line length (vessel length). Each point is the mean±s.d. of nine observations from three different mouse tumours. (**C**) Weight of tumours from mice that survived until day 14. Note, morphine treatment increased tumour growth significantly, whereas celecoxib inhibited morphine-induced increase in tumour weight. *n*=6–18 tumours.

We next examined the effect of combined treatment with morphine and celecoxib on tumour angiogenesis in tumour-bearing mice. Immunostained sections of SCK tumours from mice treated with morphine for 2 weeks showed an increase in angiogenesis (Figure 2A). Tumours in morphine-treated mice appeared to have a higher density of dilated and branching vessels as compared to PBS-treated controls. In contrast, tumours from mice treated with both celecoxib and morphine appeared to have a lower vessel density with unevenly distributed and thinner vessels, among which were interspersed hollow spaces suggestive of the absence of endothelial and tumour cells in these areas. Similarly, tumours in mice treated with celecoxib alone had small, stringy vessels with large unstained areas suggestive of the absence of endothelial and tumour cells. These qualitative observations of immunostained tumour sections were corroborated by quantitative morphometric analysis (Figure 2B). We observed increased microvessel density (CD31-PE-positive pixels, 1.4-fold), vessel number (ends, 1.9-fold), total length (1.4-fold) and branching (nodes, 2.2-fold) in morphine-treated mice as compared to PBS-treated controls (*P*=0.001 *vs* control for all values). Combined treatment of celecoxib with morphine significantly reduced all angiogenic parameters as compared to morphine by itself. Tumours in the celecoxib-treated group had lowered vessel density, number, length and branching as compared to controls, but there was no statistically significant difference. Taken together, these data suggest that morphine stimulates tumour angiogenesis in SCK tumours similar to that shown for MCF7 human tumours in nude mice (Gupta *et al*, 2002), whereas celecoxib inhibits morphine-induced angiogenesis. Importantly, celecoxib blocked morphine-induced neovascularisation in these tumours, suggesting that COX-2 plays a critical role in the tumour-promoting activity of morphine.

### Celecoxib inhibits morphine-induced tumour growth and metastasis

We next evaluated the effect of morphine and celecoxib on tumour growth in tumour-bearing mice. After 13 days of treatment with morphine, mean tumour weight in morphine-treated mice was 1.35 g compared to 0.95 g in PBS-treated controls (*P*<0.001; Figure 2C). Celecoxib significantly reduced morphine-induced tumour growth resulting in a mean tumour weight of 0.69 g compared to PBS-treated controls (*P*<0.001). Furthermore, celecoxib-treated mice showed no significant difference in their tumour weights as compared to PBS-treated controls after 13 days of treatment with celecoxib. Interestingly, tumours in morphine and celecoxib co-administered mice were significantly smaller than PBS-treated mice (*P*<0.001).

Increased angiogenesis and tumour growth after 13 days of morphine treatment was accompanied by increased metastasis (Table 1). A significant number of mice died on day 14 due to the invasiveness of this tumour model (discussed below). Therefore, we examined metastases in the lungs of mice that died on day 14 and the remaining mice that were alive after euthanasia. One hundred percent of morphine-treated mice showed metastatic foci in the lungs as compared to only 70% of control mice with lung metastases (Table 1). Lungs recovered from dead mice showed metastases in all groups except celecoxib. Of the surviving mice, 100% of morphine-treated mice showed lung metastases as compared to only 57% of PBS-treated mice. In contrast, celecoxib alone or co-administered with morphine showed metastasis in only 30 or 25% surviving mice, respectively. From these data, it appears that celecoxib prevents metastases in this model and also that induced by morphine.

### Celecoxib plus morphine increases survival

**Figure 3:**
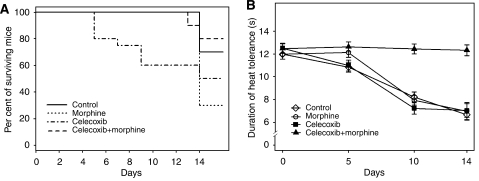
Effect of morphine and co-administration of celecoxib on survival and pain behaviour in SCK tumour-bearing mice. (**A**) Kaplan–Meier plot for survival of SCK tumour-bearing mice after treatment with morphine and co-administration with celecoxib for 13 days (after 14 days of tumour cell injection). Note, celecoxib alone led to mortality starting at day 4, whereas morphine treatment resulted in highest mortality, which occurred on day 14. Co-administration of both showed highest percent of surviving mice on day 14. *n*=20 mice/treatment. (**B**) Behavioural analysis performed for thermal hyperalgesia using Hargreave's apparatus. Right paw of the tumour-bearing leg was tested on each mouse three times at 10 min intervals for PWL. Decrease in duration of heat tolerance shows an increase in thermal perception of pain. Overall, pain increases with time during tumour growth in control as well as morphine- or celecoxib-treated mice. Co-administration of morphine and celecoxib shows the highest heat tolerance or least perception of thermal stimuli. Data are shown as mean±s.d. *n*=6–20.

We examined the effect of morphine and celecoxib on survival in tumour-bearing mice. Treatments were started 1 day after tumour cell injection and continued for 14 days. The day of death represents the number of days after the initiation of treatments. Morphine-treated mice had the worse survival rate with 70% dying by day 14 as compared to 30% of PBS-treated controls (Figure 3A). Only 20% of mice treated with morphine plus celecoxib had died by day 14, with only one death occurring by day 13. Surprisingly, 20% of mice treated with celecoxib died as early as day 4 and 40% had died by day 9, while the remainder of the mice survived until day 13 and 50% had died by day 14.

In the SCK tumour model, tumours at day 4 are barely palpable and non-metastatic. Therefore, the early death of mice after celecoxib treatment is not due to cancer progression. Several studies on rodents have used celecoxib in the range 30–250 mg per kg per mouse for several days to weeks (Koki and Masferrer, 2002; Leahy *et al*, 2002). None of these studies reported toxicity or adverse effects of these COX-2 inhibitors on the survival of mice. The only difference between our study and others is that our study used A/J mice and a SCK tumour model. It is possible that A/J mice are more sensitive to COX-2 toxicity.

To address the toxicity induced by celecoxib, we performed the same survival experiments in tumour-bearing mice with an increased initial dose of celecoxib 100 mg per kg per day on day 1 per protocol II. Surprisingly, 5 out of 10 mice receiving 100 mg per kg of celecoxib died within 24 h, and the other 5 appeared moribund (Table 2). After unsuccessful attempts to revive them, the remaining mice were euthanised after 48 h. However, mice that were co-administered morphine with 100 mg per kg of celecoxib did not show any signs of toxicity and appeared to have the same levels of activity as morphine-treated mice after 24 h. After the first day, the dose of celecoxib was reduced to 30 mg per kg per day with continued administration of 1 mg per kg per day morphine, and by day 10 of treatment (11 days after tumour cell injection), 100% of the mice were still alive. Thus, celecoxib alone impaired survival via non-cancer-related mechanisms. In contrast, morphine at higher doses was not toxic but still impaired survival at later stages by exaggerating tumour growth and metastasis. When administered together even at higher doses, morphine and celecoxib blocked the adverse effect of the other, resulting in increased survival. Taken together, these data show that a combination of morphine and celecoxib increases the survival rate of tumour-bearing mice as compared to treatment with either morphine or celecoxib alone, which is consistent with our findings that combination treatment also decreases angiogenesis and tumour burden.

### Effect of celecoxib on morphine-induced analgesia

Treatment effects on pain were tested using the PWL of the tumour-bearing leg, using a modified Hargreaves device. Baseline PWL was determined on day 0 before starting the treatments and 24 h after injecting the tumour cells. This time was chosen to avoid recording effects of pain due to the injection procedure and inflammation that could occur immediately after injection. Mice had no signs of swelling, inflammation or impaired mobility 24 h after tumour-cell injections. Baseline PWLs were the same for all treatment groups. Paw withdrawal latencies were significantly decreased in mice after 5 days of treatments (or 6 days after tumour-cell injection) with PBS and celecoxib (*P*<0.0001 *vs* baseline at day 0); both groups had palpable and measurable tumours on day 5. Morphine alone had an anti-nociceptive effect after 5 days of treatment (*P*<0.003 *vs* PBS-treated control), but had no effect after 10 and 14 days of treatment as compared to controls. In contrast to the effect of celecoxib or morphine alone, the co-administration of both resulted into a continuous analgesic effect for the entire 14 days of treatment. Paw withdrawal latencies in this group were no different than the baseline throughout the 14-day period. The duration of heat tolerance was significantly higher when both drugs were co-administered as compared to the effect of all other treatment groups (*P*<0.0001 on days 10 and 14 for morphine+celecoxib *vs* morphine or celecoxib or control). Although celecoxib treatment resulted in decreased latency *vs* control on day 10 (*P*<0.003), there was no statistically significant difference between morphine and control on day 10. On day 14, PWLs decreased further after morphine or control treatment as compared to day 10, and were no different than celecoxib treatment on day 14. Injections during the course of treatment for all groups did not result in any noticeable abnormal behaviour.

## DISCUSSION

Our study provides a proof of principle that the chronic use of opioids stimulates COX-2, leading to increased prostaglandin(s), impaired analgesia and increased tumour angiogenesis, growth, metastasis and mortality. We show that inhibition of COX-2 by co-administration of celecoxib prevents morphine-induced tumour growth and metastases and increases survival. Furthermore, co-administration of celecoxib with morphine provides analgesia even after 14 days of treatment, suggesting that celecoxib may have a salutary effect on opioid analgesia. Cyclooxygenase-2 inhibition may at least partly explain the clinical improvements in postoperative pain control that have been seen with a combination of opioids and COX-2 inhibitors and/or NSAIDs (Kroin *et al*, 2002; Malan *et al*, 2003).

Chronic administration of opioids has been linked to angiogenesis, progression of tumour growth and hyperalgesia. Tolerance to the analgesic effect of opioids is believed to be mediated at least partly by prostaglandin-mediated mechanisms (Powell *et al*, 1999). Intrathecal administration of COX-2 inhibitors attenuates the development of tolerance to morphine (Wong *et al*, 2000). Further, intrathecally administered COX-2 inhibitors have significant analgesic activity in hyperalgesic states not associated with inflammation, possibly by modulation of COX-2 that is constitutively expressed in the spinal cord (Svensson and Yaksh, 2002). Increased PGE_2_ leads to thermal hyperalgesia in the spinal cord following intrathecal delivery of protease-activated receptor (PAR)-derived peptide (Koetzner *et al*, 2004). Although the mechanisms of morphine and PAR-induced activation of PGE_2_ may be different, it appears that PGE_2_ mediates thermal hyperalgesia, irrespective of the central or peripheral site of action. Furthermore, inhibition of morphine-induced PGE_2_ by celecoxib in tumours is accompanied by prevention of thermal nociception in morphine-treated mice. It has been suggested before that nerve injury and TNF-induced pain-related behaviour is partly dependent upon peripheral prostaglandins (Schafers *et al*, 2004). Morphine stimulates TNF*α* secretion both centrally and peripherally (Gupta and Stephenson, 2007). It is therefore possible that morphine-induced upregulation of COX-2 and PGE_2_ may be due to the elevation of TNF*α* caused by morphine and/or due to some other mechanism.

The promotion of tumour growth by morphine appears to be dependent on PGE_2_-mediated stimulation of angiogenesis. Morphine-induced upregulation of COX-2 is critical in the progression of tumour angiogenesis, because tumour cell-derived COX-2 profoundly influences angiogenesis (Chang *et al*, 2004). Although endothelial COX-2 does not appear to be extremely upregulated, increased COX-2 in non-endothelial cells appears to be responsible for increased PGE_2_ in tumours of morphine-treated mice. Koki and co-workers have proposed that COX-2 is upregulated in tumour epithelial cells and stroma in response to growth factors leading to increased production of PGE_2_ that acts on endothelium and promotes angiogenesis (Koki and Masferrer, 2002). Similarly, morphine treatment stimulates COX-2 in non-endothelial cells in the tumour, which leads to increased PGE_2_ that then acts on endothelium to stimulate tumour angiogenesis. It is likely that celecoxib inhibits COX-2 in tumour cells, resulting in the inhibition of tumour growth and decreased angiogenesis. Therefore, celecoxib may inhibit angiogenesis indirectly via inhibition of tumour growth, rather than directly via anti-angiogenic mechanisms. This is further supported by observations of Koki and Masferrer (2002) that nude mice showed a decline in mammary tumour growth continuously throughout a 3-week treatment with celecoxib.

These anti-angiogenic and anti-tumour effect in rodents have been observed using extremely high doses of celecoxib. However, survival of mice was not impaired, even though animals were fed chow supplemented with extremely high amounts of celecoxib (1600 p.p.m.; ∼250 mg per kg per day) (Koki and Masferrer, 2002). Similarly, Leahy *et al* (2002) reported that nude mice treated for 40 days with celecoxib (25 mg per kg per day) had a significant reduction in tumour growth of HT-29 and HCT-116 human colon carcinoma xenografts and a reduction in the proliferation of microvascular endothelial cells). In the same study, rats implanted with pellets containing FGF2 in an intrasomal pocket in the cornea and treated with celecoxib 30 mg per kg per day for 4 or 6 days by gavage showed a significant reduction in corneal neovascularisation. In contrast to these observations, we found that mice treated with celecoxib at 30 mg per kg per day started dying 4 days after treatment. After 7 days of treatment, only 60% of the celecoxib-treated mice survived, whereas 100% of mice were still surviving in the group treated with celecoxib plus morphine. This early mortality in the celecoxib-treated group was not due to tumour growth or metastases. Administration of a higher dose of celecoxib (100 mg per kg per day) resulted in an even higher early mortality rate (50% within the first 24 h). Impaired survival in celecoxib-treated mice was therefore likely due to drug toxicity. Importantly, at both the low and high doses, celecoxib co-administered with morphine did not impair mouse survival. The survival rate in mice treated with celecoxib plus morphine was comparable to PBS-treated mice, and this rate was significantly better compared to morphine-only treatment. We believe that high doses of celecoxib used by us and others may have nonspecific activity beyond selectively inhibiting COX-2 activity. We used high doses of celecoxib, because (a) the studies described above show an inhibition of angiogenesis with the doses we used and (b) to examine if morphine could prevent high-dose celecoxib-induced toxicity. In our study, mice treated with high doses (100 mg per kg) of celecoxib and morphine survived, whereas those treated with a high dose of celecoxib alone died within 24 h of treatment, suggesting that morphine prevents high-dose celecoxib toxicity.

The loss of mice 13 days after morphine treatment appears to be related to increased tumour growth and metastasis as compared to PBS-treated mice. In contrast, mice treated with celecoxib plus morphine showed significantly reduced tumour growth as compared to PBS or celecoxib-treated mice. Immunofluorescent images of tumour sections show appreciably wider vessels in both groups of mice treated with morphine or morphine plus celecoxib. As morphine appears to induce tumour vessel dilation, we speculate that it may facilitate the availability of celecoxib in the ischaemic tumour. Earlier observations have shown that morphine induces vasodilatation by stimulating NO (Stefano *et al*, 1995). In addition, recent observations from our laboratory show that 3 weeks of morphine treatment stimulates the expression of the vasodilatory enzymes iNOS, eNOS, COX-2 and HO1 in the kidneys of C3H mice bearing NCTC 2472 tumours (Arerangaiah *et al*, 2007). Upregulation of NO by morphine may also compensate for the vasoconstrictive and hypertensive effect of celecoxib on cardiovascular haemodynamics. As COX-2 activity and NOS activity co-stimulate each other, it is likely that inhibition of COX-2 reduces NO production, resulting in haemodynamic insult. Addition of morphine may therefore prevent celecoxib toxicity by activating alternative pathways, including NOS and HO1. Irrespective of the mechanism, our data show that co-administration of celecoxib with morphine reduces tumour metastasis induced by morphine. Based on these data and supportive observations, we conclude that celecoxib and morphine may directly and/or indirectly block each other's ‘ill effects’. Therefore, although celecoxib reduces morphine-induced angiogenesis, tumour growth and metastasis, morphine prevents celecoxib-induced mortality and both together provide a better analgesic response and improved survival. It is important to note that co-administration of celecoxib with morphine resulted in significantly reduced tumour growth as well as pain relief as compared to control or celecoxib or morphine alone. Our observations are supported by another study showing that stress and morphine together reduced survival of rats bearing MAT13762B mammary ascites tumour, which was reversed by naltrexone, suggesting an opiate receptor-mediated mechanism and reduced NK cell activity (Lewis *et al*, 1983, 1984). Thus, improved survival in mice treated with a combination of morphine and celecoxib may be due to better analgesia in addition to the inhibition of angiogenesis and tumour growth.

Although our study shows that clinically relevant doses of morphine stimulate tumour angiogenesis and tumour growth and lead to reduced survival, some past studies have observed conflicting effects of morphine on angiogenesis and tumour growth. We believe that these differences are primarily due to the extremely high cytotoxic doses of morphine given in these studies as compared to clinically and physiologically relevant and opioid receptor-specific doses that ought to be administered. Ishikawa *et al* (1993) showed that 10 mg per kg morphine increases EL-4 leukaemia in C57/BL6 mice and Sarcoma 180 carcinoma in ddY mice in tumour growth in mice after 10 days of treatment. In this systematically performed investigation, morphine did not stimulate the growth of EL-4, P388, MM-46 or Meth-A cells *in vitro*. These data are in agreement with our earlier studies showing that morphine does not have any effect on MCF-7 human breast cancer cell proliferation (Farooqui *et al*, 2006) and that morphine stimulates MCF-7 breast tumour growth by promoting angiogenesis (Gupta *et al*, 2002). Morphine can affect tumour growth by (a) directly acting on the tumour cells, (b) by directly acting on the endothelial cells and (c) by acting on the central nervous system-mediated secretion of cytokines and growth factors that may alter the tumour microenvironment (Gupta and Stephenson, 2007). Morphine acts on the endothelium in a paracrine manner by increasing COX-2 activity in the tumour cells that in turn promotes tumour angiogenesis by increased PGE_2_. Therefore, morphine by itself does not modulate tumour cell growth in culture but, due to upregulation of COX-2 in tumour cells, stimulates angiogenesis *in vivo*, resulting in increased tumour growth and metastasis. These observations suggest that *in vivo* examination may be more relevant than studying the effect of morphine on isolated cells.

Conversely, some studies suggest an inhibitory effect of morphine on tumour growth. A high dose of morphine was shown to inhibit tumour growth and lung metastasis in a model of cancer pain in B16-BL6 melanoma cell growth in C57/BL6 mice, and to inhibit the adhesion, invasion and metastasis of colon cancer cells *in vitro* (Harimaya *et al*, 2002; Sasamura *et al*, 2002). Similarly, another study showed that high doses of morphine (up to 30 mg per kg) as well as naloxone inhibit MCF-7 breast cancer after d22 and MDA MB231 breast cancer progression after d14 in nude mice (last periods of observation) (Tegeder *et al*, 2003). However, the doses of morphine used in this study are not clinically relevant because humans suffering from cancer are administered far lower doses of morphine than those required for analgesia in rodents. Moreover, in MCF7 tumour model, measurable tumour growth is observed only after 2 weeks of tumour cell xenografts and differences between treatments are observed after 3 weeks of treatment, but the study was concluded on day 22. Thus, the use of clinically toxic doses of morphine in this study, as well as its early conclusion, may account for the observed inhibition of tumour growth.

Our observation of morphine-induced tumour growth is supported by earlier studies showing an inhibition of tumour growth in rodents with low dose of the OR antagonists naloxone and naltrexone, but not with higher dose (Zagon and McLaughlin, 1983a, 1983b). At low doses, naloxone antagonizes OR activity, but at higher doses, it may have nonspecific effects. More recently, the OR antagonists naloxone and naltrexone were shown to inhibit breast cancer growth in mice and rats, respectively (Koo *et al*, 1996; Tegeder *et al*, 2003). These data suggest that tumour progression may be mediated by endogenous opioids and opioids receptors acting directly on tumour cells or endothelial cells, or by other paracrine and centrally mediated mechanisms.

Some studies have demonstrated inhibition of angiogenesis by high doses of morphine and *β*-endorphin in a CAM assay (Pasi *et al*, 1991). However, these results may be due to the nonspecific activity of excessive morphine dosage, and the difference in OR presentation in the embryonic *vs* tumour endothelium. OR activity is believed to be upregulated in the tumour endothelium because of the high concentrations of pro-inflammatory cytokines and growth factors in the tumour microenvironment. Indeed, these cytokines and growth factors are known to upregulate the expression of ORs in several cell types (Gupta chapter endothelium, 2007), and growth factors such as VEGF have been shown to upregulate MOR expression in endothelial cells (Chen *et al*, 2006). As such, we believe that these findings do not contradict our hypothesis or results, but rather point to the complications of using morphine at excessive doses and in environments dissimilar to the tumour endothelium.

Our observations are in agreement with a study by Singleton *et al* (2006) that observed morphine-induced angiogenesis, using physiologically relevant doses of morphine. The authors showed that clinically relevant doses of morphine and MOR-specific agonist DAMGO stimulate angiogenesis by transactivating VEGFR2. The MOR-specific antagonist MNTX antagonized morphine-induced angiogenesis. Taken together, these observations suggest that excessive doses of morphine may inhibit angiogenesis because of their nonspecific cytotoxicity, whereas physiologically relevant doses stimulate angiogenesis via OR-mediated activity.

In conclusion, pain is closely linked to disease progression and metastasis in cancer. Therefore, finding suitable strategies to combat both without having a deleterious effect on one or the other is necessary in order to provide maximum benefit to the patient, resulting in better quality of life and improved survival. Considering that opioids are the only choice of treatment for severe pain, the addition of celecoxib to cancer-related pain management regimens may improve the analgesic response to morphine and also prevent morphine-induced angiogenesis, tumour growth and metastasis. Therefore, the hypothesis that has been proved in mice in this study warrants human trial to examine its application in the management of pain in cancer patients.

## Figures and Tables

**Table 1:** Presentation of lung metastasis on day 14 after tumour cell injection

	**Dead mice**	**Live mice**	**Total (dead+live)**	
PBS	6/6	8/14	14/20	70%
Morphine	12/12	6/6	18/18	100%
Celecoxib	0/2	3/10	3/12	25%
Celecoxib+morphine	2/2	4/16	6/18	33%

Metastasis was examined in the H&E stained lung sections of mice that died on day 14 and in the lungs of mice remaining alive. Data are expressed as number of mice with lung metastasis/number of mice in the specific treatment group on day 14 (alive and/or dead).

**Table 2:** Effect of morphine treatment on survival following high-dose celecoxib treatment

	**Percent of surviving mice after treatment**		
**Treatment type**	**After day 1**	**After day 2**	**After day 10**
(i) Vehicle control	100	100	100
(ii) Morphine	100	100	100
(iii) Celecoxib	50	0	—
(iv) Morphine+celecoxib	100	100	100^a^

After 24 h of SCK tumour cell injection, mice were treated with (a) vehicle control, 50 *μ*l normal saline+50 *μ*l 0.5% methylcellulose; (b) morphine, 0.714 mg per kg mouse per day (equivalent to 50 mg morphine per day per 70 kg human adult) during the first week, followed by 1 mg per kg mouse per day, thereafter; (c) 100 mg celecoxib per kg mouse per day for 1 day; and (d) morphine as described in (b) and celecoxib.

a 100 mg per kg mouse per day for one day followed by 30 mg per kg per day.
